# IgA vasculitis nephritis clinical course and kidney biopsy – national study in children

**DOI:** 10.1186/s12969-021-00616-z

**Published:** 2021-10-07

**Authors:** Małgorzata Mizerska-Wasiak, Agnieszka Turczyn, Karolina Cichoń-Kawa, Jadwiga Małdyk, Monika Miklaszewska, Dorota Drożdż, Beata Bieniaś, Przemysław Sikora, Magdalena Drożyńska-Duklas, Aleksandra Żurowska, Maria Szczepańska, Małgorzata Pańczyk-Tomaszewska

**Affiliations:** 1 Department of Paediatrics and Nephrology, Medical University of Warsaw, Warsaw, Poland; 2 Department of Pathomorphology, Medical University of Warsaw, Warsaw, Poland; 3 Department of Pediatric Nephrology, Jagiellonian University, Collegium Medicum, Cracow, Poland; 4 Department of Pediatric Nephrology, Medical University of Lublin, Lublin, Poland; 5 Department of Paediatrics, Nephrology and Hypertension, Medical University of Gdańsk, Gdansk, Poland; 6 Department of Paediatrics, Medical University of Silesia, Zabrze, Poland

**Keywords:** Henoch-Schönlein purpura and other vasculitides, Immunologic subjects, Clinical trials

## Abstract

**Abstract:**

The aim of the study was to investigate the relationship between the severity of typical clinical symptoms, severity of histopathological lesions in kidney biopsies in IgA vasculitis nephritis (IgAVN) and to propose indications for kidney biopsy in children.

**Material and methods:**

This retrospective study enrolled 106 patients, included in the IgAVN registry of Polish children, diagnosed by kidney biopsy. Renal and extrarenal symptoms at onset of the disease were analyzed. Biopsy results were assessed using Oxford classifications (MEST-C). The patients were divided into 3 groups depending on the severity of proteinuria: A-nephrotic proteinuria with hematuria; B-non-nephrotic proteinuria with hematuria; C-isolated hematuria.

**Results:**

The first symptoms of nephropathy were observed at the **0.7 (1–128.4) months** from the onset of extrarenal symptoms. Kidney biopsy was performed on **39 (6–782) days** after the onset of nephropathy symptoms. MEST-C score 4 or 5 was significantly more frequent in children from group A than in groups B and C. Significantly higher mean MEST-C score was found in patients with abdominal symptoms than without.

In group A: S0 and T0 we found in significantly shorter time to kidney biopsy than in S1, T1–2 *p* < 0.05) and in group B the significantly shorter time in T0 compare to T1–2 p < 0.05). The ROC analysis shows that S1 changes appear in kidney biopsies in group A with cut off 21 days (AUC 0,702, *p* = 0.004, sensitivity 0.895 specificity 0.444) T1–2 changes after 35 days (AUC 0.685, *p* = 0.022, sensitivity 0.750, specificity 0.615), and in goupn B T1–2 cut off is 74 days (AUC 0,738, *p* = 0.002, sensitivity 0.667, specificity 0.833).

**Conclusions:**

In childhood IgAVN, the severity of changes in the urine is clearly reflected in the result of a kidney biopsy. The biopsy should be performed in patients with nephrotic proteinuria no later than 3 weeks after the onset of this symptom in order to promptly apply appropriate treatment and prevent disease progression. Accompanying abdominal symptoms predispose to higher MESTC score.

## Background

IgA vasculitis (IgAV, Schönlein-Henoch purpura, HSP) is an autoimmune vasculitis characterized by the formation of IgA-dominant immune deposits in blood vessels wall [1, 2]. It occurs in both previously healthy children (90%) and, much less frequently, in adults [3]. In children, it is the most common form of vasculitis and in most cases the course is self-limiting [1, 3–5]. The incidence in children is 10–30/100000 cases per year and is the highest in autumn and winter. Peak incidence is observed between 5 and 15 years of age, in most cases < 10 years, although the literature describes cases with onset observed as early as at 6 months of age. According to most studies, the disease is more frequently observed in boys [1–3, 6–8].

The symptoms include skin lesions in the form of a hemorrhagic rash, joint pain and swelling as well as abdominal and renal symptoms. Very rarely, the disease is associated with cerebral vasculitis and nervous system disorders [3]. Usually the earliest symptoms involve skin and joints, but in some cases gastrointestinal symptoms (abdominal pain, diarrhea, vomiting) may precede the cutaneous manifestations [6, 9–12].

In 10–60% of cases, kidney involvement is observed most often between 4 to 6 weeks from disease onset; however, renal involvement may not be detected until after more than 3 months or even up to 5 years after the onset of IgAV symptoms were also reported [2, 4–6, 9, 10].

IgA vasculitis nephritis (IgAVN, HSN, Henoch-Schönlein nephritis, Henoch-Schönlein nephropathy) can manifest as microscopic or gross hematuria, proteinuria, nephrotic or nephritic syndrome, as well as acute renal failure.

The main symptom of kidney involvement is microscopic or gross hematuria, with proteinuria occurring in up to two-thirds of cases accompanied by proteinuria. The more severe the symptoms at the onset of the IgA vasculitis nephritis, the more serious the prognosis. In 30–50% of patients who develop renal lesions in the course of HSP, nephropathy becomes chronic, but only 1–7% develop end-stage renal disease [5, 7, 8, 13]. The most important factors of poor prognosis are: decreased glomerular filtration rate, nephrotic syndrome or nephritic-nephrotic syndrome, crescents in kidney biopsy and advanced lesions in kidney biopsy (ISKDC grade III-VI) [5].

The pathogenetic mechanism of the disease is not fully understood, although the key role is assigned to IgA antibodies, as evidenced by the increased serum IgA concentration observed in many patients, circulating immune complexes consisting of IgA molecules, and IgA deposits in the vessel walls of the skin and in the glomerular mesangium [3–5]. It was shown, that among the two known subclasses of IgA antibodies, vascular inflammation in HSP is caused by IgA1 with abnormal O-glycosylation in the hinge region, which under various stimuli (infections, drugs, vaccinations, etc.) form C3 containing immune complexes (also containing IgG, IgM, C1q) that are deposited in the vessels and in patients with nephropathy - in the glomeruli [1, 3, 5, 6]. Among various types of immune complexes observed in the course of HSN, IgA1-IgG complexes are characteristic solely for IgAVN, and are not observed in other forms of IgAV. Immune complexes activate an alternative complement pathway that is also implicated in disease pathogenesis; the lectin pathway is also activated through interaction between IgA and mannan-binding lectin (MBL) [1, 3, 6, 14]. In HSP patients, B cells show a defect of beta-1,3-galactosyltransferase, responsible for the attachment of galactose to the IgA1 molecule [1, 11, 14]. Due to abnormal glycosylation of the IgA1 molecule, its hepatic clearance is also reduced, which additionally increases the amount of circulating antibodies.

Renal biopsy is a key examination needed to confirm the diagnosis, determine the severity of the lesions, make treatment decisions, as well as evaluate the prognosis in IgAVN. The time to biopsy can vary and depends on the type and severity of urinary symptoms (haematuria, nephrotic/non-nephrotic proteinuria). According to SHARE recommendations and other pubications, it varies from over 4 weeks for persistent nephrotic proteinuria to up to 6 months for non-nephrotic proteinuria. But it might be even shorter in most severe cases [15–17]. Grading of histopathological lesions is based on various scores, including the five-point WHO classification and commonly used nowadays Oxford classification (MEST-C) [18–20] In the mesangial kidney biopsy, IgA deposits are found, often accompanied by C3, fibrinogen, IgG deposits, and/or IgM [1]. Typically, macrophage infiltrates in the glomeruli with IgA deposits are observed [11].

There are still no strict recommendations on the indications for kidney biopsy and time to this diagnostic procedure. Therefore we investigated the relationship between clinical symptoms with the severity of histopathological lesions in kidney biopsy in Polish children with IgA vasculitis nephritis.

## Methods

This retrospective study included 106 children (60 boys and 46 girls) seen from 2000 to 2015 with biopsy-confirmed IgAVN (HSN). The patients were hospitalized in pediatric nephrology centers and reported to the Polish Childhood HSN Registry. On the basis of the available data, the following parameters were analyzed: age at the onset of the first symptoms of IgAV, onset of extrarenal symptoms: a) skin lesions: in the form of a hemorrhagic rash located mainly on the extensor surface of the lower limbs, buttocks; b) joint involvement, i.e. swelling, joint pain, with or without limited limb mobility; c) digestive tract: abdominal pain, vomiting, diarrhea, bloody stools or a positive fecal occult blood test.

Findings of nephropathy were analyzed: the presence and intensity of proteinuria, hematuria or erythrocyturia, arterial hypertension (NT), glomerular filtration rate (GFR) and the time from the first symptoms of IgAV to the onset of nephropathy symptoms. Selected biochemical parameters were also analyzed: creatinine, albumin, IgA, complement components C3 and C4.

Proteinuria was assessed by 24 h urine collection and expressed in mg/kg/24 h: nephrotic proteinuria was diagnosed when the daily protein excretion was ≥50 mg/kg/24 h, non-nephrotic proteinuria < 50 mg/kg/24 h. The nephrotic syndrome was diagnosed in patients with nephrotic proteinuria, hypoalbuminemia < 2.5 g/dL, hypertriglyceridaemia, and hypercholesterolaemia. Nephritic syndrome was defined as the occurrence of non-nephrotic proteinuria and hematuria. Hematuria was diagnosed in the presence of > 5 red blood cells per high-power field; gross haematuria - when a noticeable discoloration of the urine was observed. Arterial hypertension was diagnosed when the mean values of systolic and diastolic blood pressure exceeded 95th per centile for sex, age, and height. GFR was calculated using the Schwartz formula [21].

Reference ranges for serum creatinine, immunoglobulin a and complement components did not differ significantly between the centers.

IgAVN was confirmed by kidney biopsy for all children studied. The kidney biopsies were assessed according to the Oxford classification by the Department of Pathomophrology in Warsaw. The following parameters were analyzed: M - mesangial hypercellularity score (M0 ≤ 50% glomeruli, M1 > 50% glomeruli); E - the presence of endocapillary proliferation: E = 0 absent, E = 1: present, S - segmental glomerulosclerosis/adhesion (S0: absent, S1: present), T - the severity of tubular atrophy/ interstitial fibrosis (T0 ≤ 25%, T1: 26–50%, T2 > 50%), C - crescents (C0 - absent, C1 < 25% of glomeruli, C2 > 25% of glomeruli).

The presence of IgA, IgG, IgM, and C3 deposits was assessed by immunofluorescence.

Patients were divided into 3 groups depending on the severity of proteinuria at the onset of nephropathy: Group A (*n* = 55) – nephrotic proteinuria with hematuria, Group B (*n* = 38) - non-nephrotic proteinuria with hematuria, Group C (*n* = 13) isolated hematuria.

### Statistical analysis

The distributions of the variables was tested with Lilliefors and Shapiro-Wilk tests. For variables with normal distributions, mean and standard deviation (SD) was calculated, for variables with non-normal distributions, median, lower/upper quartile, and min/max were calculated.Student’s t-test was used to compare the mean variables with normal distributions of the two groups; Mann-Whitney’s test was used for variables with non-normal distributions. One-way ANOVA (normal distributions) and the Kruskal-Wallis test (non-normal distributions) were used to compare more than 2 groups. Equality of variance was checked with the Levine’s test (checking the assumptions for Student’s t-test and ANOVA). The level of statistical significance was set at *p* = 0.05. The calculations were made with Statistica v.13.

## Results

The clinical characteristics of the patients is presented in Table 1.

**Table 1:** Characteristics of clinical parameters of disease onset in children with IgAVN

Parameter	
Number of patients	106
Boys/Girls	60 / 46 57% / 43%
Age of onset of HSP (years)	8.38 (2–17.25)
Skin lesions (n%)	**106 (100%)**
Gastrointestinal lesions (n%)	63 (59%)
**Joints involvement** (n%)	56 (53%)
Age of onset of nephritis (years)	8.87 (2.75–17.75)
Time from the onset of HSP symptoms to nephropathy (months)	0.7 (0–128.4)
Proteinuria (mg/kg/db)	52.75 (0–1140)
Creatinine (mg/dl)	0.54 ± 0.174
GFR (ml/min/1.73 m^2)^	126.63 ± 39.17
Albumin (g/dl)	3.57 ± 0.76
IgA (mg/dl)	217.44 ± 127.8
C3 (mg/dl)	116 ± 27.92
C4 (mg/dl)	25.03 ± 9.72

The first symptoms of nephropathy in children were observed at the age of 8.87 (2.75–17.75). The most common symptom preceding IgAVN were skin lesions, occurring in 100% of cases. Gastrointestinal complaints as well as joint pain and swelling were observed in 63 and 56%, respectively. Symptoms of nephropathy appeared 0.7 (0–128.4) months from the onset of extrarenal symptoms.

*Haematuria as a symptom was present in all patients, in addition to 52% of patients with accompanying proteinuria/nephrotic syndrome and slightly less frequently nephritic syndrome with hematuria - observed in 36% of cases. Isolated haematuria (12% of children) and gross haematuria (5% of patients) were the most rare, as shown in* Table 2*.*

**Table 2:** Clinical symptoms of IgA vasculitis nephritis

Clinical symptoms of nephritis	N	HT	↓GFR	GrossH
Nephrotic syndrome/ nephrotic proteinuria + hematuria Gr A (n%)	55 (52%)	13 (12%)	11 (10%)	2 (2%)
Non-nephrotic proteinuria + hematuria Gr B (n%)	38 (36%)	4 (4%)	4 (4%)	3 (3%)
Isolated hematuria Gr C (n%)	13 (12%)	0 (0%)	0 (0%)	0 (0%)
Sum	106 (100%)	17 (16%)	15 (14%)	5 (5%)

HT- hypertension,

GrossH- Gross hematuria

Hypertension was diagnosed in 16% of cases, mainly in children with nephrotic syndrome/ proteinuria (12%) and nephritic syndrome (4%). Reduced glomerular filtration rate was found in 14% of patients, including 10% with the nephrotic syndrome/ proteinuria, and 4% with the nephritic syndrome.

Immunological data - concentrations of IgA and the complement components C3 and C4 are presented in Table 3. The analysis was performed depending on the severity of proteinuria at the onset of nephropathy.

**Table 3:** Serum concentration of immunoglobulins and complement components

	Nephrotic proteinuria + hematuria Gr.A *N* = 55	Non-nephrotic proteinuria + hematuria Gr B N = 38	Isolated hematuria Gr C *N* = 13	P
Serum IgA (mg/dl)	213.23 +/− 100.3	252.52+/− 110.58	246.38 +/− 130.94	NS
Number of patients with elevated IgA (n%)	16 (15%)	17 (16%)	7 (7%)	NS
Serum C3 (mg/dl)	114.56 +/− 31.56	121.22 +/− 26.56	113.42 +/− 16.22	NS
Number of patients with desreased C3 level (n%)	10 (9%)	1 (1%)	0 (0%)	< 0.05*
Serum C4 (mg/dl)	23.79 +/− 9.15	26.43 +/−11.03	26.55 +/− 8.08	NS
Number of patients with desreased C4 level (n%)	9 (8%)	5 (5%)	0 (0%)	< 0.05**
Number of patients with desreased C3 and C4 levels (n%)	5 (5%)	0 (0%)	0 (0%)	< 0.05***

* A vs B, A vs C, ** A vs C, B vs C, ***A vs B, A vs C

Increased concentration of IgA was observed in 38% of children, most often in the group with isolated hematuria (group C), but the differences were not statistically significant. The decreased concentration of C3 was observed in 10% of children, significantly more often in group A (with nephrotic proteinuria) than in group B (with non-nephrotic proteinuria) (*p* < 0.05) and C (with isolated hematuria); C4 in 13% of cases. Lowered concentration of both complement components was observed in 5% of patients, only in children with nephrotic proteinuria.

**Fig. 1:**
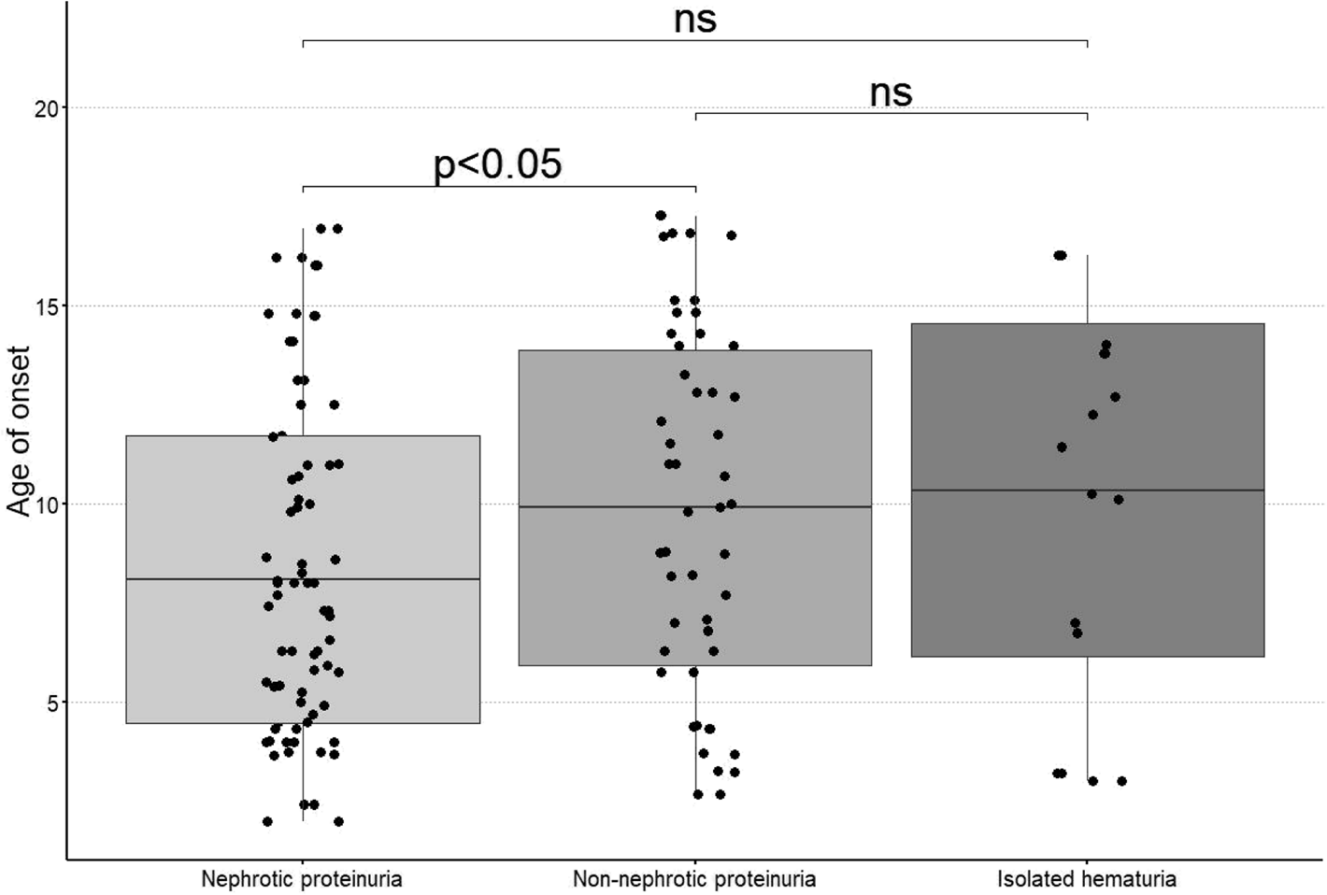
The dependence of proteinuria intensity on the age of the age of the onset th disease in children with IgAVN

A significantly lower age at onset was also demonstrated in children with nephrotic proteinuria than in non-nephrotic children, as shown in Fig. 1.

Kidney biopsy was performed *39 (6–782) days* after the onset of nephropathy symptoms, but *35 (6–782) days* in patients with proteinuria and 104 (21–358) days in patients without proteinuria. The main indication for renal biopsy was nephrotic syndrome/proteinuria. In the immunofluorescence test IgA deposits were predominant in all children *(IgA nephropathy in 100% patients),* including signs of FSGS in 8%, *mesangioproliferative* glomerulonephritis in 3%, and extracapillary glomerulonephritis in only 1%.

The analysis of the renal biopsy results was performed in groups A, B, and C, as shown in Table 4.

**Table 4:** Kidney biopsy results depending on proteinuria in children with IgAVN

	Nephrotic proteinuria Gr.A N = 55	Non-nephrotic proteinuria Gr.B N = 38	Isolated hematuria Gr C N = 13
Time to kidney biopsy from the onset **(days)**	**30 (6–111.7)**	**38 (8–758)**	**104 (21–358)**
MEST-C score			
0	1 (1%)	0 (0%)	1 (1%)
1	15 (14%)	17 (16%)	8 (8%)
2	25 (24%)	9 (8%)	3 (3%)
3	7 (7%)	10 (9%)	1 (1%)
4	4 (4%)	2 (2%)	0 (0%)
5	3 (3%)	0 (0%)	0 (0%)
> 5	0	0	0
Oxford classification			
M1	52 (49%)	36 (34%)	12 (11%)
E1	17 (16%)	5 (5%)	0 (0%)
S1	19 (18%)	9 (8%)	3 (3%)
T1	17 (16%)	7 (7%)	2 (2%)
T2	1 (1%)	0 (0%)	0 (0%)
C1	14 (13%)	15 (14%)	0 (0%)
C2	5 (1%)	1 (2.6%)	0 (0%)

According to the Oxford classification, mesangial hypercellurarity (M1) lesions were found in most patients (95%), the presence of endocapillary proliferation (E1) in 21% of patients, significantly more often in group A than in group C (*p* < 0.05); segmental sclerosis (S1) in 29% of cases, tubular atrophy/interstitial fibrosis T = 1: 25%, T = 2: 1%; C = 1: 30%, C = 2: 15% of patients.. The MEST-C score was 0 in 2% patients, 1 in 37%, 2 in 35%, 3 in 17%, 4 in 6%, and 5 in 3%; values> 5 were not observed.

**Fig. 2:**
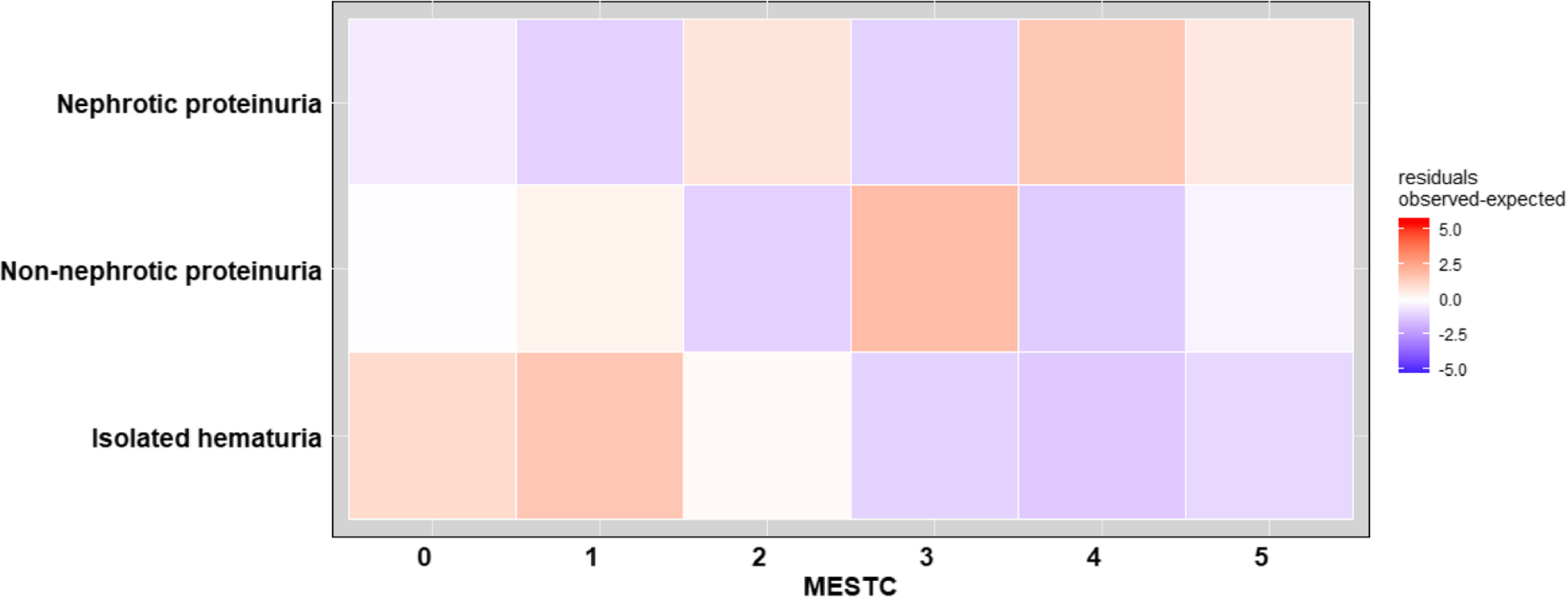
The dependence of the MEST-C score on the severity of proteinuria at the onset of the disease

The dependence of the MEST-C score on the severity of proteinuria at the onset of the disease (which were collected in a 2-dimensional array) is presented in Fig. 2. (correlogram). Correlogram shows the difference between the observed value in the study group and the expected value (if MEST-score and proteinuria were independent of each other). The correlogram is used to visualization data collected in two-way contingency table. This is residuals observed-expected MEST-C score, depends on the level of proteinuria.

MEST-C score 4 or 5 was significantly more frequent in children with nephrotic proteinuria (group A) than in groups B and C; MEST-C score 3 was significantly more often observed in children with non-nephrotic proteinuria (group B) than in the remaining groups. MEST-C score < 2 was significantly more often observed in group C.

*There were no significant differences in the mean values of: GFR, albumin, IgA, C3, and C4 between the groups*.

**Fig. 3:**
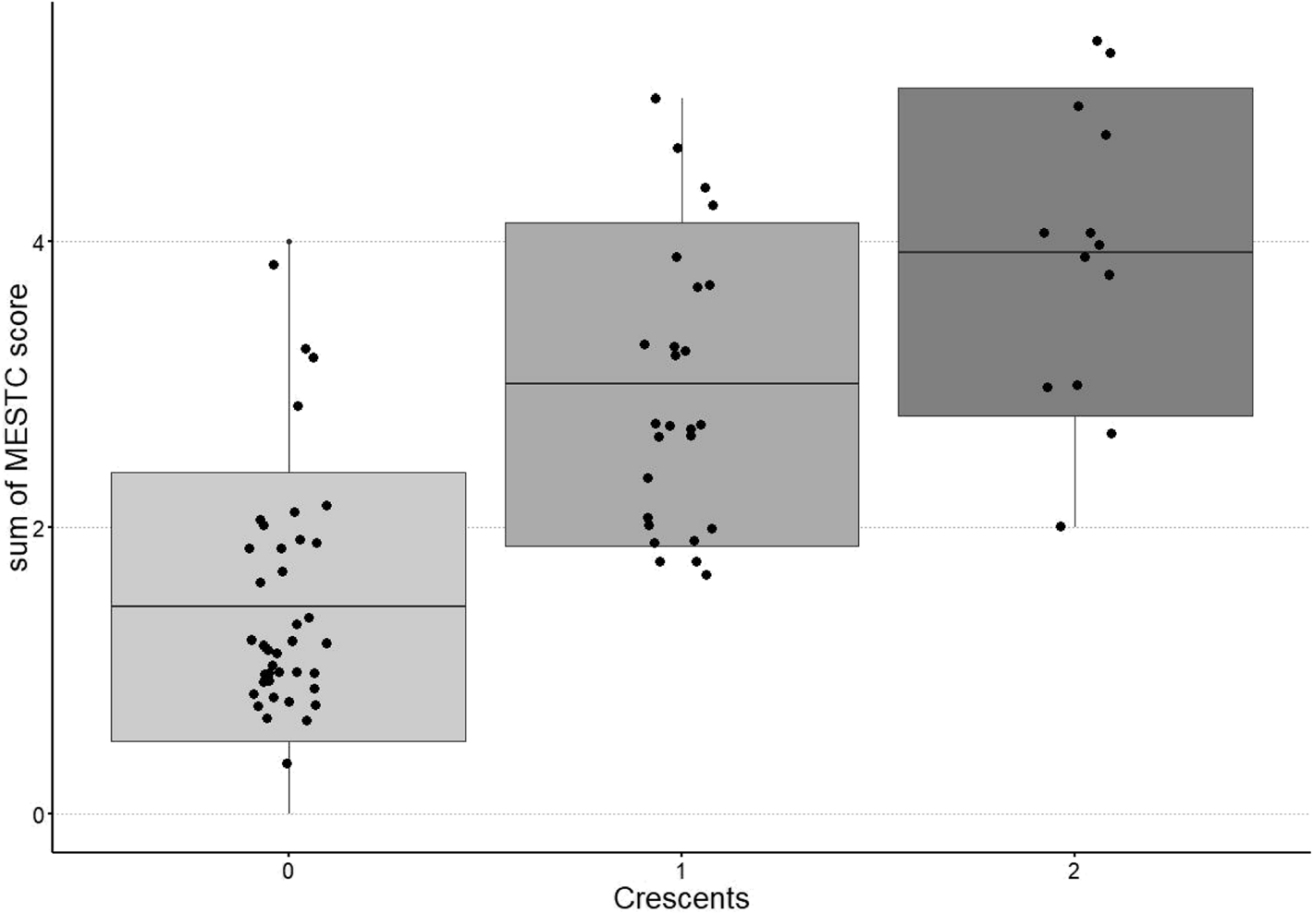
MEST-C score and crescent presence in kidney biopsy in children with IgAVN

Analyzing the results of the histopathological examination, the groups were compared depending on the presence of crescents in the kidney biopsy: C0, C1, C2. The only differences observed were significantly lower MEST score in the C0 group than in C1 and C2, as shown in Fig. 3. The remaining mean values of biochemical parameters, including proteinuria and GFR, did not significantly differ between the groups.

In respect to MEST-C score, the following groups were analyzed: MEST-C < 2, MEST-C score 2–3 and MEST-C score > 3. MEST-C score > 3 group had significantly higher proteinuria values than MEST-C score < 2 (*p* < 0.05).

Significantly higher mean MEST-C score was observed in patients presenting abdominal symptoms than in the group without gastrointestinal symptoms (*p* < 0.01).

*In children with nephrotic proteinuria and S0 and T0 we found significantly shorter time to kidney biopsy than in S1, T1–2 (25 (6–782)* vs *52 (17–584) days and 24(6–782)* vs *43 (11–584) days, respectively, p < 0.05); in group non-nephrotic proteinuria the significantly shorter time in T0 compare to T1–2 was observed (median 33 (8–522)* vs *104 (22–3308) days, p < 0.05).*

**Fig. 4:**
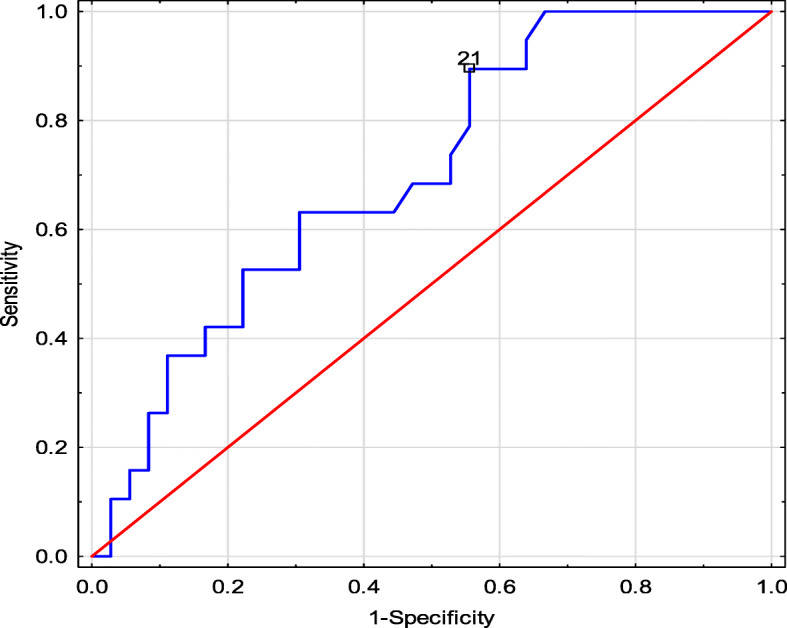
ROC analysis: Sensitivity and specificity of the time to renal biopsy at which S1 occurs in children with nephrotic proteinuria

*Therefore, we performed ROC analysis to determine the cut off time to renal biopsy after which S1 and T1–2 appear in children with nephrotic proteinuria and T1–2 - in children with non-nephrotic proteinuria. The ROC analysis shows that S1 changes appear in kidney biopsies in children with IgAVN and nephrotic proteinuria after 21 days (AUC 0,702, p = 0.004, sensitivity 0.895 specificity 0.444, as shown in* Fig. 4*), T1–2 changes after 35 days (AUC 0.685, p = 0.022, sensitivity 0.750 specificity 0.615), and in children with non nephrotic proteinuria T1–2 cut off is 74 days (AUC 0,738, p = 0.002, sensitivity 0.667 specificity 0.833).*

The presence of immunoglobulin deposits and complement components in kidney biopsy was also analyzed.

Isolated IgA deposits were significantly more often observed in group C than in groups A and B (*p* < 0.05). IgM deposits were significantly more frequent in group A and B than in C (p < 0.05). Deposits containing IgA, IgG, and IgM were present only in group A and B.

## Discussion

In our retrospective study, we analyzed the relationship of renal and extrarenal IgAV (HSP) symptoms with the result of kidney biopsy classified in Oxford classification in patients with IgAVN (HSN), diagnosed in 2000–2015 and reported to the registry. For this purpose, we analyzed the course of the disease and the results of kidney biopsy in 106 patients with confirmed IgAVN.

Calvo-Rio et al. reported the following frequency of extrarenal symptoms: skin lesions 97.6%, gastrointestinal symptoms 64.5%, and joint involvement 63.1% of cases [22]. Similarly, in the study by Johnson EF et al. gastrointestinal symptoms were observed in 65% of people, and joint involvement in 68% [23]. In our analysis, skin lesions were detected in 100% cases as the first symptom, gastrointestinal lesions in 59%, and joint lesions - in 53%. According to the literature, extrarenal lesions such as abdominal pain, bloody stools, and persistent skin lesions constitute an independent risk factor for the development of HSN [1, 4, 7]. Our study, however, confirmed a significantly higher MEST-C score in kidney biopsy in patients with abdominal symptoms.

The time between the onset of nephropathy and the first extrarenal symptoms in our group was 0.7 (0–128.4) months, which is consistent with the data reported by other authors [1, 8, 11]. In one patient, this period was 128.4 months, i.e. about 10 years, which is the longest time described in the literature from the onset of HSP symptoms to the onset of nephropathy.

In the meta-analysis preformed by Narchi H et al., including 1133 patients, hematuria was the most common manifestation of HSN, occurring in 78%, nephrotic and nephritic syndromes were less frequent and concerned 21% of cases [9], and hypertension was reported in 19% of patients [24]. The latter differs from our studies in which nephrotic proteinuria or nephrotic syndrome and nephritic syndrome were the dominant symptoms of nephropathy, diagnosed in 52 and 36% of children, respectively. Isolated hematuria was the rarest and it concerned 12% of patients, which may be due to the indications for a kidney biopsy in this group of children. These results show that the renal manifestation of IgAV can vary widely.

The incidence of decreased concentration of complement components in the serum is similar in different studies and amounts to 7–9% on average. In our study, it was 10–13%. According to the data in the literature, no correlation has been found between the degree of decreased concentration of complement components and the presence of renal lesions and their severity [2, 25, 26]. In our study, decreased concentration of C3 was observed significantly more often in children with nephrotic proteinuria than without, and the simultaneous decrease in C3 and C4 level occurred only in children with nephrotic proteinuria- in 5% of cases.

The study was performed in a polish Childhood HSN (IgAVN) Registry cohort, in a population with biopsy-confirmed diagnosis. Although the Registry gathers information on all polish IgAVN pediatric patients, the number of cases is limited by the fact that not all pediatric nephrology centers have performed a kidney biopsy due to changes in urine or it was performed only in the most severe cases.

In our study, the time to kidney biopsy was 39 (6–782) days, which is similar to the Finnish authors and slightly longer than the French authors, where the median was 21 (10–39) days [17, 19]. The application of the WHO classification made it possible to show the relationship between the severity of lesions in kidney biopsy and the time of sampling.

Mesangial IgA deposits were found in all patients. As evaluated according to Oxford classification, mesangial proliferation was found in 95% of biopsies, endocapillary proliferation (E1) in 21% of patients, sclerosis in 29%, tubular atrophy/interstitial fibrosis 26%, and crescents in 45% of children. These data are similar to the study by French authors, except endocapillary proliferation, which was found in 86% of cases [17].

Due to the MEST-C score being the lowest < 2 in the group with isolated hematuria (MEST-C score = 1 was associated with M1), the presence of proteinuria should be an indication for renal biopsy, as also indicated by the correlogram shown.

The severity of proteinuria at the onset correlated with MEST-C score in the kidney biopsy, and the presented figure (correlogram) can be used to visualization the result of the relationships between histopathological examination and proteinuria is a very useful result of our work.

Similarly, the presence of crescents was found in patients with higher MEST score.

Very important in our work is significantly shorter time to kidney biopsy in children with nephrotic proteinuria and S0 vs S1 (median 25 vs 52 days) and T0 vs T1–2 (median 24 vs 43 days) and also with non-nephrotic proteinuria and T0 vs T1–2 (median 33 vs 104 days). In our opinion, the time to renal biopsy in children with proteinuria, especially nephrotic proteinuria, should be shortened to the shortest possible time, associated with the treatment of concomitant infections, mainly bacterial, if present. Our study shows, that chronic lesions S1 appers from 21 day after onset of nephrotic proteinuria, therefore we recommend kidney biopsy in this group of IgAVN children up to 3 weeks. In children with non nephrotic proteinuria cut of for T1–2 is 74 days. This recommendation differs from the Consensus, which recommends renal biopsy if nephrotic proteinuria persists > 4 weeks [16]. To date, IgAVN is known to be an acute inflammatory process that requires appropriate treatment [27].

The limitation of this work is its retrospective character.

## Conclusion

In childhood IgAVN, the severity of changes in the urine is clearly reflected in the result of a kidney biopsy. The biopsy should be performed in patients with nephrotic proteinuria no later than 3 weeks after the onset of this symptom in order to promptly apply appropriate treatment and prevent disease progression. Accompanying abdominal symptoms predispose to higher MESTC score.

## Data Availability

The datasets analysed during the current study available from the corresponding author on reasonable request.
